# Opportunities and challenges of whole-genome and -exome sequencing

**DOI:** 10.1186/s12863-017-0479-5

**Published:** 2017-02-14

**Authors:** Britt-Sabina Petersen, Broder Fredrich, Marc P. Hoeppner, David Ellinghaus, Andre Franke

**Affiliations:** Institute of Clinical Molecular Biology, Kiel University, Kiel, Germany

**Keywords:** Whole-genome sequencing, WGS, Whole-exome sequencing, WES, Next-generation sequencing, NGS, Complex diseases, Inflammatory bowel diseases, Variant priorization, Variants of unknown significance (VUS)

## Abstract

Recent advances in the development of sequencing technologies provide researchers with unprecedented possibilities for genetic analyses. In this review, we will discuss the history of genetic studies and the progress driven by next-generation sequencing (NGS), using complex inflammatory bowel diseases as an example. We focus on the opportunities, but also challenges that researchers are facing when working with NGS data to unravel the genetic causes underlying diseases.

## Background 

Studies of human genetic variation using DNA sequencing have undergone an extraordinary development from their introduction over 40 years ago up to current technologies, which allow for a human genome to be sequenced and analyzed within a matter of days at consumable costs of approximately 1000 USD. The first widely used method was developed by the British chemist Frederick Sanger in the 1970s [1] and he received the Nobel prize in 1980. “Sanger sequencing” relies on nucleotide-specific chain-terminating inhibitors to identify the sequence of a specific fragment of DNA. The method was continuously refined over the years and incorporated in the first generation of automated sequencers. Sanger sequences show very high accuracy but are restricted to a single DNA fragment at a time and a maximum sequence length of 1000 bp. In addition to the low throughput, high costs render this technology unsuitable for routine large scale sequencing projects. The largest effort using the Sanger technique was the Human Genome Project with the goal of identifying the complete sequence of the human genome [2], which is in essence based on different donors from Buffalo (New York, USA) [3, 4]. Completion of the project took over a decade (1990–2003), involved more than 20 institutes all over the world and cost nearly 3 billion dollars. Still, for many years, Sanger sequencing was the prevailing technique to identify causative mutations in monogenic diseases. However, the limitations of the technology meant that finding the one gene responsible for a disease was tedious work. Rather than performing large-scale, indiscriminate sequencing, numerous experiments were often necessary to narrow down candidate regions from microsatellite-based linkage studies and pinpoint to one or a few candidate genes that would then be sequenced. In most cases, these experiments required samples from large pedigrees with several affected individuals to successfully identify candidate regions small enough for further analysis. These issues are further amplified in the study of genetically heterogeneous diseases with causative variants in a number of genes or very large genes, as well as diseases that do not follow a Mendelian inheritance pattern, but instead have a complex genetic background involving tens to hundreds of genes. The common disease-common variant hypothesis assumes that a large part of the heritability of these complex diseases can be attributed to variants with a minor allele frequency above 1% (single nucleotide polymorphisms, SNPs) in the general population, each variant having a small additive or multiplicative effect on the disease phenotype. Addressing questions as complex as these clearly required novel approaches.

However, it was not until the introduction of high-throughput genotyping in the early 2000s, enabling the interrogation of several hundred thousand to millions of genotypes in thousands of cases and controls [5], that genome-wide association studies (GWAS) became a reality. For the first time a quick and unbiased screening of SNPs throughout the whole genome was possible, thereby facilitating the detection of susceptibility regions for complex diseases. What followed can be referred to as the “GWAS era”, with genome-wide case-control association studies carried out for numerous complex diseases, identifying more than 25,000 significantly disease-associated genetic loci until today [6]. GWAS studies primarily focused on common SNPs, excluding rare variants. Later approaches like Illumina’s human exome genotyping array [7] shifted the focus to include rare, exonic variants. However, it soon became clear that genotyping efforts alone were not sufficient to completely uncover the genetics behind complex diseases [8].

The release of the first “next-generation” sequencing instruments (NGS; see [9] for an overview) in the mid-2000s led to a first revolution in disease study, offering vastly improved speed at significantly lower cost - enabling the generation of a whole human genome sequence in a matter of weeks for 10,000 USD by 2011 [10]. In addition to price and performance, the new sequencing technology also proved to compensate for some of the technical weaknesses of the older sequencing and genotyping technologies, allowing for the genome-wide detection of variants, including novel ones, at a low cost. However, despite the immense drop in sequencing costs for a human genome, large-scale sequencing projects were still costly and therefore not yet carried out for thousands of samples as routinely done in GWAS.

In 2007, Craig Venter published the first diploid genome sequence of a single individual, which was created using the gold-standard Sanger sequencing technology, and which is perhaps still among the most accurate and best-annotated human genomes released to the public domain [11]. However, DNA materials of the donor are, to our knowledge, not available to the public for benchmarking and follow-up studies. This year however, the academic Genome in a Bottle Consortium provided extensive NGS data on seven genomes, including two trios, which serve as open benchmarking data and materials [12].

The next breakthrough for NGS in human genomics arrived with the introduction of targeted enrichment methods, allowing for selective sequencing of regions of interest [13] and thereby dramatically reducing the amount of sequences that needed to be generated. The approach is based on a collection of DNA or RNA probes representing the target sequences in the genome, which can bind and extract the DNA fragments originating from these targeted regions. Whole exome sequencing (WES), which enables sequencing of all protein-coding regions in the human genome (the exome) quickly became the most widely used targeted enrichment method, especially for monogenic (“Mendelian”) diseases. This approach enabled the detection of both exonic (coding) as well as splice-site variants, while requiring only approximately 2% of sequencing “load” compared to whole genome sequencing (WGS). The unbiased analysis of all genes eliminates the need for a time-consuming selection of candidate genes prior to sequencing. It has been estimated that the exome harbors about 85% of mutations with large effects on disease-related traits [14]. In addition, exonic mutations were shown to cause the majority of monogenic diseases [15], with missense and nonsense mutations alone accounting for approximately 60% of disease mutations [16]. While these numbers may be in part biased by the difficulty of identifying disease-causing mutations in non-coding regions, the success of exome sequencing studies for monogenic diseases confirms its advantages. In the years following its introduction, exome sequencing led to a vast increase in the identification of Mendelian disease genes [17, 18]. This is reflected for example in almost 2000 new entries in OMIM since 2008 (current total: 4787), describing the molecular basis of a particular phenotype.

Current large-scale genome and exome sequencing projects [19–22] have not only provided crucial information on variant frequencies in different populations, but have also shown that a human genome typically contains an estimated 100 genuine loss-of-function variants, completely inactivating around 20 genes [23]. Therefore, sequencing of healthy individuals or representative population samples can also lead to important insights into disease. Focusing on seemingly “healthy human knockouts” can aid in detecting the true effects of variants previously assumed to be disease-causing [24] and exploring gene function in general, thus elucidating the “resilience” phenomenon further [25].

In recent years, NGS has also been increasingly applied for addressing pharmacogenomic research questions. It is not only possible to detect genetic causes that explain why some patients do not respond to a certain drug, but also try to predict a drug’s success based on genetic information [26]. Certain genetic variants can affect the activity of a particular protein and these can be used to estimate the probable efficacy and toxicity of a drug targeting such a protein [27]. NGS therefore has applications far beyond finding disease-causing variants. For inflammatory bowel diseases (IBD) we refer to the exhaustive pharmacogenomics review of Katsanos and colleagues [28].

### Progress of genetic research for common complex diseases

Some of the diseases that profited immensely from GWAS are inflammatory bowel diseases. Together with ulcerative colitis (UC), Crohn’s disease (CD) is one of the two main sub-phenotypes of IBD. IBD are chronic, relapsing disorders involving inflammation of the gastrointestinal tract, sometimes accompanied by extra-intestinal manifestations. The disease onset can occur at any age, but the peak for CD as well as UC is in early adulthood (approximately 25 to 35 years of age). In the clinic, symptoms include chronic flare-ups of inflammation, abdominal cramping pain as well as diarrhea and weight loss, thereby greatly affecting the quality of life of patients. In Europe, an estimated 1.4 million people suffer from CD [29] but as of yet, there is no known cure and the current treatment is solely aimed at controlling the symptoms. The current consensus is that IBD is caused by the complex interplay of an overly active immune system and environmental triggers (such as bacterial infections, dietary habits or smoking) in genetically susceptible individuals [30, 31]. The strong genetic component, especially for CD, is reflected by familial clustering of disease occurrence and a concordance of 35% in monozygotic but only 3% in dizygotic twin pairs [32]. The relative risk for developing IBD is estimated to be 15 times higher for first degree relatives of an IBD patient than in the general population [33].

**Fig. 1:**
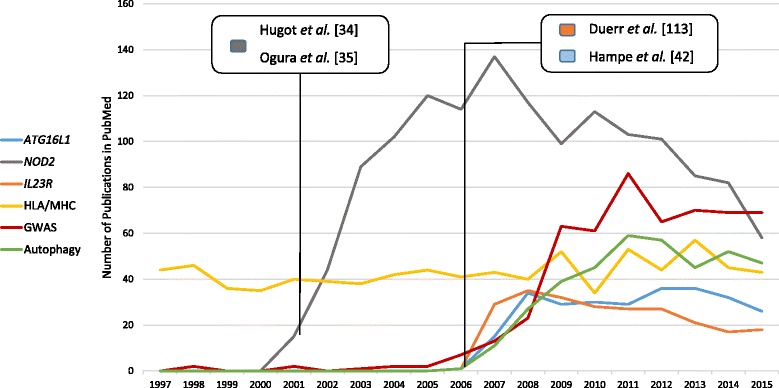
Number of PubMed citations for *ATG16L1*, *NOD2*, *IL23R*, HLA/MHC, GWAS and autophagy in combination with “inflammatory bowel disease”, “Crohn’s disease” OR “ulcerative colitis” from the years 1997–2015 depicting a steep increase of follow-up studies for genes and pathways after discovery. Interestingly, the HLA/MHC association signal in IBD has been known for a long time, however, studies for this locus in IBD are rarer and no increase can be observed. We think that this region is understudied, given its importance in disease etiology (in particular in ulcerative colitis), calling for more IBD immunogenetics studies in the future

Due to the complex nature of IBD, genetic research focused on the identification of genetic risk factors that increase the susceptibility to the disease, typically common SNP alleles that are significantly more frequent in patients than in healthy controls. The aforementioned methods have all contributed to the discovery of genetic risk factors for IBD in the past. Genome-wide linkage and candidate gene studies during the late 1990s were able to identify the first susceptibility loci for IBD through positional cloning and candidate gene analysis. The first susceptibility gene to be identified for CD was *NOD2* [34, 35], encoding for a member of the cytoplasmic nucleotide-binding oligomerization domain (NOD)-like receptor (NLR) protein family. Over the years, several association studies added significantly to the number of identified loci [36, 37], followed by meta-analyses which combined the data of several individual GWAS-studies from all over the world. The larger sample sizes led to more statistical power and eventually to the discovery of numerous additional susceptibility loci [38–40]. Today, more than 200 loci have been identified for IBD [41] and have highlighted some key pathways involved in the etiology of IBD. Figure 1 illustrates the success of hypothesis-free genome-wide studies. For example, our group first unveiled the link of autophagy to IBD by identifying *ATG16L1* in a genome-wide candidate SNP screen [42]. Before, *NOD2* had been identified as the first and so far best-replicated Crohn’s disease susceptibility gene through two independent studies [34, 35]. Gene identification is then ideally followed by numerous validation and in particular functional/mechanistic studies of the respective candidate genes. Bringing disease genes on the radar of the research community leads to further studies, then scientific publications (as shown by the steep increase of publications per year in Fig. 1) and ultimately an improved disease understanding. However, the variants identified in GWAS still explain less than 30% of the estimated genetic variance of IBD [40]. While IBD constitutes perhaps one of the greatest success stories and role models in complex disease genetics research, GWAS have also been quite effective for a number of other complex diseases like psoriasis [43–45], atopic dermatitis [46, 47] and primary sclerosing cholangitis (PSC) [48, 49]. Combined analyses of several of these immune-mediated diseases have even revealed considerable overlap of susceptibility loci, pointing at true pleiotropy and shared disease etiologies beyond CD and UC, while also showing complex disease-specific patterns at shared loci as well as revealing disease-specific loci [50, 51].

Interestingly, the genetic susceptibility factors for IBD are, with a few exceptions (e.g. *NOD2*, *TNFSF15*, *HLA*), the same in European-ancestry and East-Asian IBD patients [41]. Similar results have been obtained for other complex inflammatory diseases, such as systemic lupus erythematosus [52]. Therefore, trans-ethnic studies will clearly aid in identifying consistent genetic risk loci for complex inflammatory diseases, thus increasing also statistical power due to the larger sample sizes. The few differences in the genetic risk maps may also help in pinpointing likely existing different environmental factors in the countries under study.

**Fig. 2:**
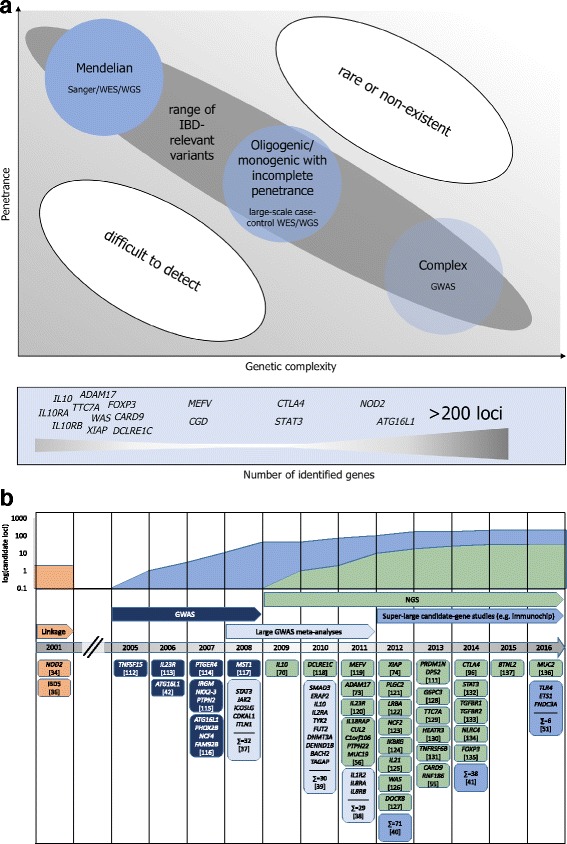
**a** Top: Range of IBD-relevant variants based on genetic complexity underlying the disease and variant penetrance. Bottom: Overview of identified IBD genes ranging from monogenic to complex forms based on the highest known penetrance for each gene. For both *NOD2* and *PRDM1*, for example, both common and rare variants have been identified as disease-relevant in patients [110, 111]. **b** Timeline of gene discovery for IBD [112–137]. Top graph shows cumulative number of genes separated by technology (log scale)

As previously indicated, GWAS studies focused on SNPs with moderate to high allele frequencies in the general population. A part of the so-called missing heritability may however be found in rare variants with larger effect sizes [53] for some diseases. Results of a recent large-scale sequencing project of more than 2600 genomes and almost 13,000 exomes did not support the idea that lower-frequency variants have a major role in predisposition to type 2 diabetes [54]. For IBD, however, common and rare susceptibility variants have been shown to even coexist in the same genes, as is the case for *NOD2* [34, 55, 56]. Figure 2a illustrates this wide range of IBD-relevant variants concerning their penetrance and the genetic disease complexity and provides an overview of the identified genes from monogenic, fully penetrant genes to those harboring common susceptibility variants. Rare and especially novel variants can best be detected by DNA sequencing and the development of NGS finally made this feasible for complex diseases. Figure 2b depicts the discovery of IBD genes since 2001 employing the different technologies discussed here and shows the great success of GWAS on the one hand, but also the increasing relevance of NGS during the past few years.

### Application of NGS for complex diseases

The usage of NGS and especially exome sequencing for Mendelian disorders proved to be extremely successful. Even sequencing of just a single patient could lead to the discovery of the genetic mutation responsible for the disease by filtering the detected variants based on functional consequence (e.g. missense, nonsense, splice-site variants) and allele frequency in the general population, for example in the data of the 1000 genomes project [19, 57] or the Exome Aggregation Consortium (ExAC) [20]. But when dealing with complex diseases, different approaches need to be considered.

One possibility is the application of the GWAS approach to NGS data, aiming for the identification of significant differences between cases and controls. Disease-associated common variants can best be detected by GWAS and sequencing approaches have the potential to complement this by discovering rare variant associations, given that the necessary large sample sizes are considered. However, with a decreasing allele frequency, the power to detect genes or variants of interest also decreases, if effect sizes are small to moderate. Single marker association testing is therefore often “underpowered” for rare variants with frequencies below 0.5% or even 0.1% minor allele frequency (MAF), since the number of observations of such alleles is often not large enough to achieve statistical significance due to small sample sizes [58]. For example, observing an allele once with 0.5 or 0.1% MAF with 99% probability requires sequencing of at least 460 or 2300 individuals, respectively. Assuming a disease-associated variant with 0.1% MAF and an allelic odds ratio (OR) of 1.4, the sample size (cases and controls with equal sized groups) required to achieve 80% power is 540,000, given a disease prevalence of 5% and a significance level of 5 × 10^−8^ [59]. However, the commonly used significance level of 5 × 10^−8^ is valid for approximately one million common tag SNPs (MAF ≥ 5%) only if a linkage disequilibrium *r* ^2^ < 0.8 for pairs of tag SNPs is applied. With 0.1% MAF, we would need a *P*-value threshold level of 1 × 10^−8^ and 3 × 10^−7^to meet genome-wide and exome-wide significance (at *r* ^2^ < 0.8), respectively [60]. Several statistical methods have been proposed in the past to perform case-control studies with WES or WGS data, most of them using variant aggregation approaches to address this issue. The two main types of aggregation tests comprise burden and variance component tests [58] or a mixture of both. Burden tests [61, 62] compare the number of variants in a certain region or gene between cases and controls, while variance component tests (e.g. the sequence-based kernel association test, SKAT [63]) can distinguish between protective and risk variants in a single gene, making them more powerful if the gene possesses a mixture of protective and risk variants. Lee et al. [59] provide a comprehensive overview of currently available algorithms.

The successful application of these tests is however limited by sample size, as sequencing studies involving WES or WGS still require a significantly larger sample size than a typical GWAS to identify significant rare variant associations [64]. Despite continuously decreasing prices for sequencing, case-control studies employing thousands of individuals still remain a costly undertaking compared to GWAS where the latest array generation (e.g. Global Screening Array from Illumina with 700,000 variants) is currently available for less than $40 per sample. The NGS approach is therefore still restricted to large-scale sequencing centers, companies, healthcare providers (e.g. Geisinger Health), consortia involving several institutes and crowd-sourced approaches.

Therefore, the focus currently lies on the analysis of unusual cases to find highly penetrant variants. Here, one possibility is sequencing of large families with several affected individuals to narrow the dataset down to few candidate variants based on those shared by the affected individuals [65]. Clustering of patients within a pedigree may point to variants with larger effects on disease compared to those identified in GWAS and even monogenic forms of IBD. However, it can also simply indicate the accumulation of a large number of common susceptibility alleles in the pedigree [65] and exome sequencing may therefore not necessarily be successful. Apart from multiplex families, the most informative characteristics indicating the presence of a highly penetrant genetic cause are an early age of onset and very severe course of disease. GWAS performed specifically for pediatric IBD (age of onset <18 years) failed to clearly distinguish early onset from adult IBD, identifying known IBD loci or exclusive pediatric loci that were later also identified for adult IBD [66, 67]. There is great overlap between susceptibility genes identified for pediatric and adult-onset IBD (more than 30 loci described [66]). Early-onset cases of IBD, with a disease manifestation during the first 10 years of life, often show a more severe disease course with a higher risk of complications and a higher frequency of indeterminate colitis (IC) diagnoses [68]. Patients classified as very-early-onset even develop the disease during the first 6 years of life. A large spectrum of monogenic diseases, mainly immunodeficiencies, can also present with IBD-like intestinal inflammation [69]. However, several studies have also identified shared genetic factors underlying these monogenic, early-onset and adult-onset IBD cases with rather oligogenic or polygenic causes. Mutations in genes for the interleukin 10 receptor (*IL10R*) subunit proteins [70] and the *IL10* gene itself [71] were shown to be responsible for several cases of severe early-onset IBD (eoIBD). At the same time, *IL10* was also associated with adult-onset UC [72] and CD [39] in GWAS. Other identified causes of eoIBD include a deletion in *ADAM17* (ADAM metallopeptidase domain 17) [73] and mutations or deletions in the *XIAP* (X-linked inhibitor of apoptosis) gene [74–76] in male patients. Although the direct overlap between key genes associated with IBD and IBD-like monogenic disorders is rather low, the affected proteins often interact directly or indirectly with each other and share common signaling cascades that contribute to IBD etiology [69]. Results from monogenic forms therefore have the potential to give important insights into mechanisms contributing to disease. An excellent overview of the genetics of early- and very early-onset forms of IBD is the review by Uhlig et al. [77].

Targeted resequencing of susceptibility regions has also been applied for several immune diseases and has identified additional rare, functional variants in susceptibility genes, which were detected using common variants in GWAS. For instance, gene resequencing for atopic dermatitis identified low-frequency missense variants in the *GARP* gene as significant contributors to disease risk [78]. Perhaps not surprisingly, monogenic disease forms of complex diseases—i.e. patients that carry variants with very high penetrance—have not exclusively been detected for IBD, but also for other diseases. For example, monogenic forms of psoriasis caused by mutations in *CARD14* [79] were revealed through exome sequencing of a family with early-onset psoriasis. Studies of rare variants in Mendelian forms of disorders that are symptomatically similar to systemic lupus erythematosus (SLE) have highlighted pathways also playing a role in the complex disease form. As another example, *TREX1*, encoding for the three prime repair exonuclease 1, has been associated with monogenic Aicardi-Goutières syndrome [80], a disease displaying phenotypic overlap with SLE. More recently, 0.5% of SLE patients were shown to also harbor mutations in this gene [81].

While sequencing of severe early-onset patient exomes greatly facilitated the identification of novel, high penetrance variants, their discovery among the tens of thousands of variants identified in an exome is still a major challenge. Since the first exome studies that relied on allele frequencies from the 1000 genomes pilot [82], several large-scale sequencing studies for genomes and exomes have been undertaken. Databases like EVS [83], ExAC [20] and KAVIAR [84] now provide population-specific allele frequencies from several thousands to more than 60,000 individuals that can be used for filtering of candidate variants. However, some of these databases are “contaminated” with data from patients or yet unknown patients of similar symptoms as the disease of interest, so the data should be used with caution.

The interpretation of non-coding variants has proven to be extremely challenging. The ENCODE project [85] significantly facilitated the understanding of functional elements in the human genome. However, the complex analysis of these sites is not yet routinely carried out in most projects. For exome data, the analysis of non-coding variants is limited from the beginning, due to the nature of the technology with exclusive enrichment of exons and, in some cases, UTRs. Variants from exomes therefore tend to be reduced to those that are most likely to affect protein structure. Nonsense, start-loss, stop-loss and splice-site variants as well as frameshift insertions and deletions (InDels) have rather clearly defined effects on the protein and are present in comparably low numbers. The interpretation of sometimes hundreds of rare missense variants represents a greater challenge. Several *in silico* prediction tools are available to identify those amino acid changes that most likely affect protein structure. SIFT [86] and Polyphen-2 [87] were the first widely used tools, more recently DANN [88], CADD [89] and FATHMM [90] were introduced. The latter promise improved accuracy and additionally offer predictions for non-coding variants. Other tools specifically focus on identifying splice-altering variants, including those located farther away from the exon-intron boundary [91, 92]. Genes also differ concerning the amount of potentially disruptive genetic variation they can tolerate, expressed for example by the residual variation intolerance score (RVIS) [93]. The prediction of the effect of a variant on the protein structure and thereby its function is however only one of the levels that need to be considered when aiming to detect disease-relevant variants. Variants diminishing the function of a gene do not necessarily manifest as an observable phenotype. This can for example be due to redundancy of the function in several genes, preventing the deficiency of one from having an effect. Filtering and priorization of variants based on these criteria can already significantly reduce the number of candidates. In some cases, this is sufficient to identify a likely causative variant relying on the known function of a well characterized gene or novel variants in a known disease gene. In most cases, however, additional filtering is needed. In general, it is helpful to analyze more than one individual of a family, even when dealing with sporadic cases, since this allows the identification of variants segregating with the disease within the pedigree. For sporadic cases the healthy parents can also be used to detect *de novo* mutations in the patient. These filtering steps can, however, still result in a number of variants remaining, without being able to clearly identify the most likely candidate. Novel genes that haven’t previously been implicated in disease or even genes with an unknown function substantially complicate the search. The question then arises, how to proceed with a handful of candidates with a possible but unconfirmed pathogenic effect (variants of unknown significance, VUS) that remain after filtering with all available methods. Functional analyses, especially for genes that are not yet well characterized, can be time-consuming and expensive.

The case of a family with Crohn’s disease and autoimmunity in two children [94] nicely illustrates this issue. Exome sequencing was performed and yielded several candidates, among them a rare missense variant in *CTLA4* (Cytotoxic T lymphocyte-associated protein 4). While it represented a likely candidate, it was also present in the asymptomatic mother. This incomplete penetrance, as well as other candidate variants, made interpretation and priorization difficult. Also, heterozygous CTLA-4 deficiency in mice does not induce a phenotype [95], which made the role of the detected variant in disease questionable. Additional evidence pointing to *CTLA4* finally emerged when variants were also identified in other patients with immune phenotypes [96, 97] and functional studies were able to back the role of heterozygous *CTLA4* variants in immune dysregulation. The incomplete penetrance suggests that additional modifying factors yet need to be revealed, requiring the analysis of additional patients with CTLA-4 deficiency.

### Developing infrastructure for data sharing

Reliably classifying disease-causing variants often involves finding correlations between different, independent observations, i.e. patients or cohorts with similar clinical phenotypes in which the same (or a functionally related) variant has been observed. For very rare or private variants only a second patient with the same symptoms and the same genetic variant is sufficient for statistical proof of the original finding. Sources of information are usually published studies and public data repositories that need to be searched, manually or with specifically set up local bioinformatics pipelines. However, the complexity of the data at hand (including sometimes dozens of VUS for larger patient cohorts) as well as the vast amount of sequences that is now routinely being generated and deposited, is calling for more efficient and integrated approaches.

Several efforts exist that aim to specifically aggregate relevant clinical data, including databases such as Decipher [98], HGMD [99] or ClinVar [100]. Complementary to these resources, projects are under way to better link national infrastructures and communities. Of note here are, for example, the Belgian “SymBioSys” (http://www.kuleuven.be/symbiosys/) or the German “VarWatch” project (BMBF project ID01EK1506 [101]), both targeting separate issues in the integration of NGS data and clinical variants. The main goal of SymBioSys is to leverage national NGS data and provide efficient access. It does so by building a federated network across sequencing facilities, together with a generic interface that helps in rapidly mining the data for identical variants or study parameters. VarWatch, on the other hand, is focused directly on the clinical context and is designed to function as both a repository and a “monitoring” tool. Clinicians can submit their VUS, together with phenotypic information about the disease, and VarWatch will continuously search for matching cases, both within its own data repository as well as external resources.

**Fig. 3:**
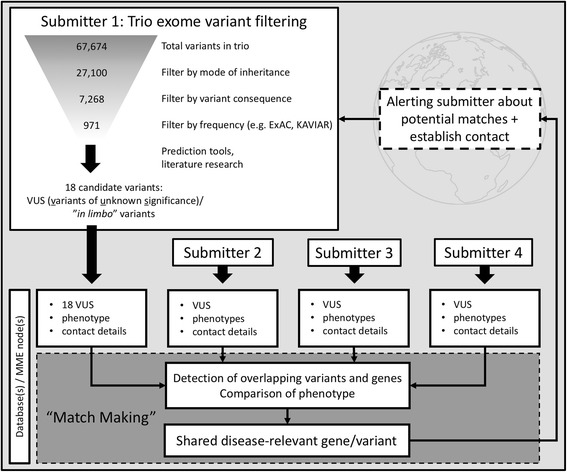
Course of a typical trio exome project yielding several VUS and benefit of MME for variant selection. Filter by mode of inheritance: recessive or dominant; by variant consequence: missense, nonsense, splice-site, start-loss or stop-loss; by frequency: maximum minor allele frequency of 1% in various databases (ExAC, EVS, in-house controls)

While these initiatives are potentially important building blocks towards generating comprehensive clinical resources, they leave the larger issue of how to efficiently access and integrate the globally accumulating information about the genetics of individual patients and their conditions unanswered. A solution that is finding strong support amongst larger databases and bioinformatics institutes is currently being developed by the “Global Alliance for Genomics and Health” (GA4GH), an international consortium of clinicians and bioinformaticians with the goal of providing standards and software for sharing clinical data on a global scale. One product of these activities has been the “Beacon” network, and in extension “MatchMaker Exchange” (MME) [102]. The focus of Beacon and MME is to provide a “connective tissue” between various “information islands”, linking databases through a common interface and enabling simple, platform agnostic queries without having to create huge aggregations of data. Databases connected to the beacon network can easily be queried for the presence of specific variants. MME further extends this concept, allowing users not only to find identical variants, but also to include information about the clinical context of the variant (such as observed phenotype). In doing so, it can bring together clinicians and researchers with patients whose variants are not strictly identical, but potentially related on a functional level and thus further help finding diagnoses. Figure 3 depicts the variant filtering of one real-world example from our clinic for trio exome sequencing. While the filtering steps are able to reduce the number of variants from more than 67,000 to only 18 variants potentially of interest, it is still difficult to select the best candidate among these VUS or *in limbo* variants. One possible solution for this problem is the usage of MME which can detect overlaps between the VUS submitted by different scientists or clinicians and establish contact between them, making it possible to pinpoint the causative variant(s) and thus solve the clinical case (statistical significant result through recurrent finding of very rare event).

It is also becoming increasingly clear that in addition to efficient access to distributed variant information, there is also a growing need for metadata standards to describe clinical observations not only genetically, but also phenotypically. While several vocabularies have been proposed over the years, the ones in use – such as the Unified Medical Language System (UMLS) [103] - are focused more on syndromes and less so on the symptoms a patient is presenting with. This information however will likely be vital, especially when trying to match rare variants and rare diseases with poor representation in standard nomenclatures. A promising solution has recently been proposed in the form of the Human Phenotype Ontology (HPO) [104], a collection of hierarchical, phenotypic descriptors organized in an ontological network similar to the already well-established sequence ontology (SO) [105] and gene ontology (GO) [106]. In addition to providing a complementary nomenclature for clinicians to better characterize their findings, the inherent network-like structure of the HPO also allows to measure the distance between any two terms. This enables more complex matching scenarios, for examples when clinicians have used slightly different but related terms or sets of terms to characterize their patients [107].

### Future directions

#### Combination of methods

Apart from genome and exome sequencing, which we focus on here, there are several other NGS applications that we expect to increase in relevance as efforts are concentrating on linking observed mutations to functional consequences beyond putative coding changes. Of great interest here are the detection of modulation in gene activity, for which both the direct sequencing of transcripts through RNAseq as well as the detection of differentially methylated sites (DMS) by means of bisulfite sequencing as proxy for regulation hold great promise. A completely different but equally important line of inquiry is the metagenomic sequencing of the host-associated microbiome to detect possible correlations between the presence or absence of certain genera and disease, as has already been suggested for a decrease in Bacteroides and Firmicutes and a reduced diversity of the microbiota in IBD patients [108]. The combined application of these multi-omics data has the potential to provide an improved overall picture of the characteristics of a certain disease and therefore help to understand its molecular underpinnings. With sequencing costs further decreasing, large case-control studies with sample sizes comparable to GWAS are also slowly becoming a reality and will help detect rare variant associations specifically for complex diseases. The biggest challenge though remains, identifying relevant environmental factors in complex diseases. Genetics and other functional genomics analyses may also help in hinting at the disease-causing environmental factors.

#### Technological developments

WES and WGS allow for the accurate identification of single-nucleotide variants (SNVs) and small InDels. For the detection of large InDels, copy number variations (CNVs) as well as genomic rearrangements, however, deep sequencing and meticulous analyses are needed, which are mostly not yet part of the common analysis pipelines used in the majority of projects.

NGS is already being applied to the clinic for the diagnosis of certain diseases, mostly through deep sequencing of gene panels. However, relevant variants still require confirmation through Sanger sequencing due to the generally lower quality of NGS data, so it is desirable to further increase the quality of NGS in the near future.

New methods are continuously being developed to use NGS for additional applications or to extract more information from standard applications. 10X Genomics for example offers an additional instrument (Chromium), which is fully compatible with the workflows of available NGS sequencers and enables large-scale phasing of variants and structural variant detection from WGS and even WES as well as single cell applications by generating synthetic long reads. The Chromium instrument uses emulsion to partition DNA. Barcoding and amplification of smaller fragments from the original larger fragments then takes place in droplets called “GEMs” that include all necessary reagents, resulting in the small fragments stemming from one larger molecule carrying the same barcode. These “synthetic long reads” can therefore be linked over larger regions of the genome. The workflow delivers ready-to use libraries for sequencing and software for the analysis and visualization is openly available.

Other companies opt for the development of new sequencing technologies, often called “third-generation sequencers”. Pacific Biosciences performs single molecule, real-time (SMRT) sequencing of DNA fragments using immobilized DNA-polymerases and produces reads of over 10,000 bp average length. Nanopore sequencers, like those developed by Oxford nanopore, detect the DNA sequence of a single-stranded DNA molecule by passing it through a protein pore and measuring a shift in voltage that originates from interactions with the pore. However, these single-molecule technologies are still too expensive and not yet applicable for resequencing larger numbers of complete human genomes.

Looking further into the future, several exciting new technologies are on the horizon. Genia Technologies, which was bought by Roche in 2014, is currently developing a nanopore-based sequencing technique with a focus on diagnostic applications. First results have already been published [109], showing promising proof-of-principle results. However, it will likely still take several years until the method is ready for the market. Illumina is planning the launch of a new semiconductor sequencer in 2017 as part of their Project Firefly, but as of yet, no details have been released to the public. GenapSys by Sigma Aldrich promises a low-cost, portable sequencer with a purely electronic sequencing chip, but more information is currently only available to its testers.

## Conclusions

The extraordinary progress in the development of methods for genomic analysis during the past 15 years and especially the breakthrough in NGS in the past decade has led to an enormous increase in the understanding of the human genome and its relation to disease. Improved technologies continuously provide faster, cheaper and more accurate results, allowing us to move from gene panels to exomes to routinely sequencing whole genomes in the clinic in the near future. It has however become increasingly clear, that to make the most of the large, complex datasets being generated, scientists must work together more than ever, to achieve the ultimate goal of translating genomic data into clinically actionable results that patients can directly profit from. With the generation of genomics data continuously becoming easier and cheaper, the interpretation of the large amounts of data and the identification of the relevant disease-causing environmental factors will remain the biggest challenges of the years to come.

## Abbreviations

CD: Crohn’s disease
CNV: Copy number variation
DMS: Differentially methylated site
ExAC: Exome Aggregation Consortium
GA4GH: Global Alliance for Genomics and Health
GO: Gene ontology
GWAS: Genome-wide association study
HPO: Human Phenotype Ontology
IBD: Inflammatory bowel disease
InDels: Insertions and deletions
MAF: Minor allele frequency
MME: MatchMaker Exchange
NGS: Next-generation sequencing
OR: Odds ratio
PSC: Primary sclerosing cholangitis
RVIS: Residual variation intolerance score
SKAT: Sequence-based kernel association test
SLE: Systemic lupus erythematosus
SMRT: Single molecule, real-time
SNP: Single nucleotide polymorphisms
SNV: Single-nucleotide variant
SO: Sequence ontology
UC: Ulcerative colitis
VUS: Variants of unknown significance
WES: Whole exome sequencing
WGS: Whole genome sequencing
